# Associations between Food Pantry Size and Distribution Method and Healthfulness of Foods Received by Clients in Baltimore City Food Pantries

**DOI:** 10.3390/ijerph18136979

**Published:** 2021-06-29

**Authors:** Yuxuan Gu, Shahmir H. Ali, Sally Yan, Bengucan Gunen, Reuben Park, Lisa Poirier, Hope C. Craig, Hengjin Dong, Joel Gittelsohn

**Affiliations:** 1 Center for Health Policy Studies, Department of Social Medicine, School of Public Health, Zhejiang University School of Medicine, Hangzhou 310058, China; guyuxuan@zju.edu.cn; 2 Center for Human Nutrition, Department of International Health, Johns Hopkins Bloomberg School of Public Health, Baltimore, MD 21205, USA; bgunen1@jhu.edu (B.G.); lpoirie4@jhmi.edu (L.P.); hcraig2@jhmi.edu (H.C.C.); jgittel1@jhu.edu (J.G.); 3 Department of Social and Behavioral Sciences, School of Global Public Health, New York University, New York, NY 10003, USA; sha371@nyu.edu; 4 Family League of Baltimore City, Baltimore, MD 21218, USA; sally.yan25@gmail.com; 5 Krieger School of Arts and Sciences, Johns Hopkins University, Baltimore, MD 21218, USA; rpark21@jhu.edu

**Keywords:** food pantry, food insecurity, nutritional quality, systems, Baltimore, client choice

## Abstract

This study aimed to evaluate the association of the overall nutritional quality and the weight share of specific types of foods received by food pantry clients with food pantry size and distribution method. Data on healthy food weights using the gross weight share (GWS) of select foods and the validated Food Assortment Score Tool (FAST) were collected from 75 food pantry clients in Baltimore, Maryland. The average FAST score across the study population was 63.0 (SD: 10.4). Overall, no statistically significant differences in average FAST scores by pantry size and distribution method were found. However, among client-choice pantries, clients of small pantries had higher scores (*p* < 0.05) while among medium pantries, clients of traditional pantries had higher scores (*p* < 0.01). Subgroup analysis of GWS was stratified by pantry size and distribution methods. Findings suggested multi-level, multi-component interventions combining environmental strategies are needed to enhance the healthfulness of foods received by clients. Our analysis provided data to consider further refinements of pantry interventions and planning of more rigorous research on factors influencing the effectiveness of pantry interventions.

## 1. Introduction

Food insecurity was experienced by 11.1% of United States (U.S.) households in 2018, including households in large urban centers such as Baltimore city [1]. According to Feeding America, about 23% of people residing in Baltimore currently experience food insecurity, including more than 30,000 children [1]. In Baltimore, there are over 220 food pantries working with the Maryland Food Bank [2]. Roughly half of these organizations are operated by volunteers at community-based nonprofit organizations such as churches and homeless shelters, whereas the other half are located within Baltimore City Public Schools. Clients of food pantries tend to be food insecure and may be vulnerable to nutritional deficiencies [3]. The rise in obesity and diet-related diseases among food insecure individuals in the U.S. brings to question the nutritional quality of foods accessible to households that rely on food pantries [4]. Additionally, the most recent updates to international food guides have also confirmed the importance of dietary healthfulness [5,6,7].

Feeding America and many food banks in the United States, including the Maryland Food Bank, present the client choice method as a best practice. The main motivation for promoting client choice is to provide a dignified experience to clients. Pantries were classified by distribution method: traditional (distributing pre-packed bags) or client choice (allowing clients to make selections on the foods they receive). Additionally, client-choice can be implemented using a number of different models: the supermarket model (clients can shop like at a store), table model (food items/groups are displayed on tables), inventory list model, and food weight model (clients can select a set poundage of food), among others. A study using a Freshplace intervention included a client-choice pantry in the north end of Hartford and increased fruits and vegetables by one serving per day compared with the traditional group [8]. However, it is not clear whether client choice influences the overall healthfulness or share of specific groups of client food selections. If it is found that a switch to client choice is independent from the availability of healthy foods in client bags, food assistance organizations will need to take additional measures beyond promoting client choice to promote healthy options to their clients. In addition, food pantry size may also matter while considering the healthfulness of foods clients receive. Pantries of different sizes may have different capacities and resources. For example, larger pantries may have more resources like fridges which are good for healthier foods including fresh fruit and vegetables to be stored. Pantry size may also matter in developing environmental strategies to try and nudge healthier client selections because we can see if different intervention strategies like client-choice or not will have different impacts in different-sized food pantries. It is also unclear whether the pantry size differentiates the healthfulness of client food selections.

The main objective of organizations like food pantries are to minimize chronic hunger. However, the managers of food pantries we spoke with in our previous studies said they were also interested in stocking and providing healthier foods if they knew clients were interested in using them, but perceived barriers associated with it [9].

Previous studies have assessed the quality of food distributed in food pantries [10,11,12,13]. At the pantry level, the quality and quantity of foods accessible to clients may be determined by the food distribution method used in the pantry [14]. In urban food pantries in the Bronx, NY, USA the nutritional quality of foods available varied by item type (fresh, shelf-stable, refrigerated/frozen), sourcing, distribution method (prefilled bags and client choice), and client position in line. They found that client choice pantries in Bronx, NY, USA had healthier foods available than traditional pantries. However, at client choice pantries, earlier clients selected the less healthy options first. This suggests that a switch to client choice or stocking healthy foods alone might not be sufficient to promote healthy options to clients [10]. However, the generalizability of these limited findings in other US settings remains unclear. Likewise, no previous studies have investigated the association with many food pantry characteristics such as pantry size and food distribution method together with nutritional quality of foods received by clients [13]. Thus, in this study, we evaluated the association of the overall nutritional quality and the weight share of specific types of foods received by food pantry clients with food pantry size and distribution method. We assumed that both food pantry size and distribution method (separately or combined) would affect the healthiness of food clients get. By including sociodemographic factors, we also wanted to see if, in a client choice pantry, clients from certain sociodemographic backgrounds selectively preferred healthier foods. In the pantries distributing pre-packed bags, we wanted to see if pantries serving certain sociodemographics were more likely to try to distribute healthier foods. This information would help us identify clients who typically do not get healthier foods at pantries and target them in our intervention messaging in the future.

## 2. Methods

### 2.1. Study Population

This study used baseline data from a feasibility trial to promote healthy foods and beverages in Baltimore City food pantries [15]. Data collection was conducted in seven food pantries in Baltimore, Maryland during September and October of 2018. We identified 102 food pantries using the Maryland Food Bank’s database of partnering Baltimore community food pantries located outside of Baltimore City Public Schools. Indeed, this descriptive formative research study was nested under a larger interventional study that iwas occurring at the randomly selected pantries that were analyzed. Pantries were stratified into tertiles of size, based on pounds of food distributed in the previous fiscal year: small (65 to 10,000 pounds), medium (10,001 to 24,600 pounds), and large (24,601 or more pounds). Exclusion criteria for food pantries included (1) operating less than once per week, (2) being located in a school, (3) already receiving nutrition education from the Maryland Food Bank or the University of Maryland Extension, and/or (4) having a new manager (<2 months in position). Of the pantries in the Maryland Food Bank’s database, 21 food pantries could not be reached, 58 were not eligible, and 9 were not interested; 7 food pantries of differing sizes (determined by weight of food distributed in the previous fiscal year) were randomly selected out of 14 eligible and interested food pantries.

**Figure 1:**
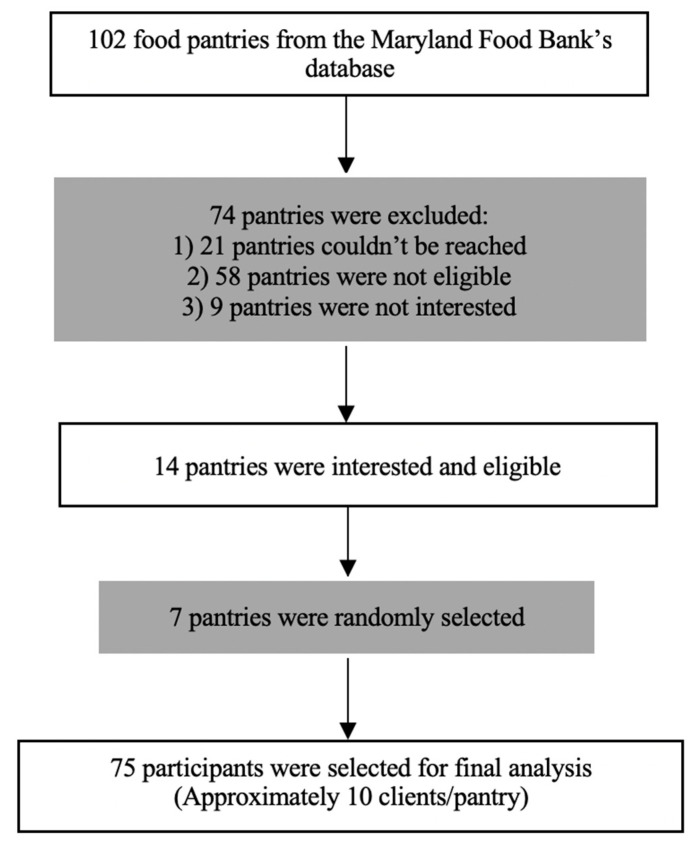
Flow chart of participants in this study.

Food pantry clients were recruited during food pantry distribution hours and they were be provided with a 10-dollar Walmart card after completing the whole survey. Clients participated in an on-site survey and data of 75 participants were collected by trained research assistants (approximately 10 clients/pantry, a convenience sample, in which the first 10 clients approached and who agreed to participate comprised the sample). Eligibility criteria for participants included being at least 18 years old and receiving food from the pantry at the time of data collection. The flowchart of the study is shown in Figure 1.

### 2.2. Measurements

A food pantry client questionnaire was developed for this study, which had 11 questions including a client bag audit and sociodemographic information, which takes approximately 15 min. The questionnaire was validated and tested in our formative research. The survey was enumerated by research assistants. Client-sourced data were important in the case of our study as (1) we were interested in the specific foods selected by each client (rather than general information on foods in the inventory in the pantry), and (2) food pantries often did not keep a record on which foods each client selected, thus necessitating us to actively source the data ourselves. The overall nutritional quality of the foods received was assessed using the Food Assortment Scoring Tool (FAST) developed by Caspi et al. (2018) [12]. A higher FAST score reflects a greater proportion of healthy foods. The FAST measure was developed with 13 scored categories and 31 sub-categories. The FAST scores were generated by sorting and weighing food in categories, multiplying each category’s weight share by a healthfulness parameter, and summing the categories (range 0–100). The FAST tool has been previously validated against the Healthy Eating Index 2010 (HEI-2010) in food pantry setting [12]. Gross weight share (GWS) is the proportion of the client’s selection that is allocated towards a food category. To calculate the GWS of certain food groups, the weight of a proportion of select food groups was extracted from the FAST score calculations and separately analyzed. Sociodemographic information collected included age, sex, ethnicity, household information, marital status, employment, food assistance program participation, and medical history. We collected this information because, in Baltimore City, the availability of healthy foods in the food environment is closely associated with sociodemographic factors [1].

### 2.3. Statistical Analysis

A descriptive quantitative analysis of pantry clients’ sociodemographic information was conducted and stratified by food pantry distribution method and pantry size. Analysis of variance (ANOVA) was used to assess significant differences by group. The final analytic sample included 74 participants who completed all parts of our questionnaires. All analysis was performed in R Version 3.5.3 (https://www.r-project.org/, available on 29 June 2021).

## 3. Results

### 3.1. Description of Pantry Client Characteristics and FAST Scores

Among our sample, there were four traditional pantries and three client-choice pantries. An overview of participant characteristics is displayed in Table 1. The mean age in years of the participants was 56.6 years old. Of the participants, 90% were self-identified as African American. Most clients lived in households of one or two individuals (52.7%) and lived in households without children (58.1%). More than half of clients received SNAP benefits (57.5%), but few received WIC benefits (5.6%). In terms of self-reported medical history, most reported high blood pressure (62.2%), followed by diabetes (25.7%), obesity (13.5%), and cancer (8.1%).

### 3.2. Associations between Food Pantry Characteristics and FAST Scores

The average FAST score across the study population was 63.0 (SD: 10.4). There was no difference in FAST scores by food pantry size or by distribution method, overall (Table 1). Clients who received WIC benefits had lower FAST scores compared with clients who did not receive WIC (−12.2, *p* < 0.05). No other sociodemographic variable was significantly associated with FAST scores for the overall sample (Table 2).

Sub-group analysis was limited by the relatively small number of participating pantries. However, in medium-sized food pantries, clients from the client-choice pantry received food bags with lower FAST scores compared with clients from pre-packaged pantries (*p* < 0.01). In pre-packaged pantries, the healthiness of client bags was not statistically significantly different among different sized pantries. Among client-choice pantries, food selections received by clients from medium pantries had lower FAST scores, followed by large and small pantries (*p* < 0.05) (Table 3).

### 3.3. Analyses of GWS Stratified by Pantry Size and Distribution Methods

When stratified by pantry size, clients of small pantries had higher GWS of fresh fruits and vegetables (*p* < 0.01), higher GWS of beverages (*p* < 0.001), vegetable protein (*p* < 0.01), and mixed meals and side dishes (*p* < 0.001) compared with clients of medium and large pantries. Medium pantry clients had higher GWS of processed fruits and vegetables (*p* < 0.01) and higher condiments, baking, and cooking (*p* < 0.01) compared with clients of small and large pantries. Large pantries’ clients had higher GWS of highly processed meat (*p* < 0.05), desserts and snacks (*p* < 0.05), dairy (*p* < 0.001), and meat, poultry, fish, and eggs (*p* < 0.001) than small and medium pantries’ clients.

When stratified by distribution methods, client-choice pantries’ clients had higher GWS of beverages (*p* < 0.001) and condiments, baking, and cooking than traditional pantries’ clients (*p* < 0.01) (Table 4).

## 4. Discussion

This is the first study to explore whether food pantry distribution method and size can predict the healthfulness of food obtained by pantry clients in an urban setting. Increasingly, attention is being directed to the client choice model and the need for a healthier food environment for all pantry clients. In client choice pantries, clients are not required to receive items they may already have, may not like, or cannot eat for health or personal reasons, which may decrease food waste and be more efficient for food distribution [8,16]. In a past study, a food pantry intervention that involved transforming a traditional pantry to client choice saw improvements in client food insecurity and fruit and vegetable intake [17].

Overall FAST scores were not significantly different among clients of different sized pantries and between clients of pantries having different distribution methods, suggesting no noteworthy differences in healthiness of foods clients received by pantry size and distribution methods founded in our study. Thus, the client-choice method may not significantly improve the nutritional quality of foods received by clients compared with traditional prepackaged methods in these seven pantries. However, outside of simply nutritional outcomes, such a method may still catalyze other positive outcomes, such as less food waste and better connection with clients, which may also have salient ramifications for nutrition and health outcomes of food pantry clients.

Among client choice pantries, clients of small pantries received healthier foods than clients of large pantries, followed by medium pantries. This suggests that the size of client-choice pantries impacts nutritional quality of foods received by clients. Small food pantry clients received the largest proportion of healthy foods. In addition, medium and large food pantries often distribute more foods and serve more clients than small ones. These findings suggest clients who visit larger food pantries may have the greatest potential to benefit from nutrition programs and interventions in pantry settings.

Clients of client-choice pantries obtained foods of significantly lower nutritional quality than clients of traditional pantries for medium-sized pantries. This suggests that, although clients are able to make their own food selections in the client choice model, the foods available in the pantry food environment and clients’ nutritional knowledge and motivation remain important determinants of the healthfulness of the client bag [13]. From our formative research [9], the pantry managers using the traditional distribution method we spoke with did not purposefully try to push healthier foods to clients in pre-packed bags. The reasons for preferring pre-packed bags were more related to maintaining safety, order, respect in the pantry, and for serving clients as quickly as possible. Additionally, not all client-choice pantries offered true client choice by allowing clients to select whatever they wanted, but some of them had restrictions as to the types of foods that could be selected. It’s important to note that not all client-choice pantries offered the same level and flexibility of choice.

In terms of the proportional weight of select food groups received by food pantry clients, clients across food pantries of different sizes and distribution methods received a significant portion of both healthy and unhealthy foods. In other words, clients received a variety of both fresh fruit and vegetables, beverages, desserts, and snacks, which suggests food pantries are offering a diverse range of foods. Nonetheless, while healthy food options were indeed received, this study supports past research suggesting there is still room to significantly enhance the proportion of healthy foods for food pantry clients [10]. In our intervention studies, we were aiming to promote lean and low-sodium proteins, fruits and vegetables, and healthy carbohydrates.

This study had several limitations. Chief among these was the generalizability of the results due to sampling issues. The sample size was small, participation was low among pantries invited to participate, and the sample selection of pantries and of clients was not random. Nevertheless, our analysis provided data to consider further refinements of pantry interventions and planning of more rigorous research on factors influencing the effectiveness of pantry interventions. This also limited us from doing some regression analyses to examine if certain client characteristics were predictors of the nutrition quality of foods received. For example, if the pantry used a client-choice model, their clients’ disease history or health status may have influenced their food selection. The power of the factors studied in our study explaining the differences in healthier foods obtained by pantry clients were limited by this study’s low effect modification. These results as preliminary descriptive information require confirmation in a larger study. In particular, the client choice method does not appear to be a panacea for ensuring higher nutrition quality foods are selected, further intervention is needed to promote healthful selection. Second, we used FAST to determine the healthiness of clients’ food, instead of the Healthy Eating Index (HEI) [18], to better target food pantry settings. Although FAST is correlated with HEI-2010 scores [12], the HEI is commonly used to report findings on other food sources (supermarket and corner store purchases, school meals, etc.) and diets, thus limiting our ability to compare results with studies considering other sources of food for low-income, food-insecure individuals [12]. Third, clients taking more nutritious food home from a pantry does not necessarily imply a healthier diet. For many clients, pantries supply only a portion of their overall food supply. However, for clients who usually visit pantries, those food are a very important part of diets for their daily lives. In addition, the eligibility criteria were more suitable to selecting pantries for our intervention study, which may reduce the generalizability of this sample.

## 5. Conclusions

The nutritional quality of foods that clients receive at food pantries may impact clients’ diet and be associated with health outcomes. While average FAST scores across clients from pantries of various sizes and distribution methods did not show statistically significant differences, stratifying FAST scores in subgroup analysis and examining GWS of key food groups suggested that clients from larger food pantries received the largest proportion of unhealthy foods. These findings suggest the need to prioritize larger food pantries in future interventions, and likewise increase clients’ nutritional literacy to facilitate healthy food choices. Multi-level, multi-component, and combined interventions including environmental strategies, distribution system changes, and clients’ nutritional intention and behavior on food pantries are needed to improve the nutritional quality of food pantries serving food-insecure communities in the US.

## Figures and Tables

**Table 1:** Sociodemographic characteristics and FAST scores of clients (*n* = 74) by food pantry size and distribution methods.

	Total	Food Pantry Size			Food Distribution Methods	
	(*n* = 74)	Small (*n* = 21)	Medium (*n* = 20)	Large (*n* = 33)	Traditional (*n* = 45)	Client-Choice (*n* = 29)
Overall (%)	100	28.4	27.0	44.6	60.8	39.2
Age (in years) mean (SD)	56.61 (14.34)	50.81 (12.48)	59.65 (14.45)	58.45 (14.70)	56.42 (14.50)	56.90 (14.33)
Female (%)	55.4	66.7	65.0	42.4	55.6	55.2
Race (%)						
Black/African	89.2	90.5	90.0	87.9	93.3	82.8
Others	10.8	9.5	10.0	12.1	6.7	17.2
Household Size (Number of people) (mean (SD))	2.81 (1.62)	3.76 (1.61)	2.45 (1.32)	2.42 (1.58)	2.73 (1.64)	2.93 (1.60)
Number of Children in household (mean (SD))	0.85 (1.27)	1.43 (1.50)	0.50 (0.76)	0.70 (1.26)	0.78 (1.26)	0.97 (1.30)
SNAP ^1^ = yes (%)	57.5	71.4	55.0	50.0	61.4	51.7
WIC ^2^ = yes (%)	5.4	10.0	5.0	3.1	6.8	3.6
Marital Status (%)						
Married	8.1	9.5	10.0	6.1	6.7	10.3
Unmarr. w/part.	12.2	14.3	10.0	12.1	11.1	13.8
Unmarr. w/o part.	73.0	61.9	80.0	75.8	77.8	65.5
Other	6.8	14.3	0.0	6.1	4.4	10.3
Employment (%)						
Empl. 30+ h/wk	10.8	23.8	5.0	6.1	15.6	3.4
Empl. <30 h/wk	5.4	4.8	10.0	3.0	4.4	6.9
Seasonally empl.	1.4	4.8	0.0	0.0	0.0	3.4
Unemployed	18.9	14.3	10.0	27.3	17.8	20.7
Retired	27.0	0.0	40.0	36.4	26.7	27.6
Disabled	36.5	52.4	35.0	27.3	35.6	37.9
High blood pressure (%)	62.2	66.7	70.0	54.5	55.6	72.4
Diabetes (%)	25.7	14.3	45.0	21.2	26.7	24.1
Obesity (%)	13.5	9.5	5.0	21.2	17.8	6.9
Cancer (%)	8.1	14.3	10.0	3.0	13.3	0.0
FAST score (mean (SD))	63.04 (10.38)	65.49 (6.79)	62.62 (7.39)	61.75 (13.37)	63.92 (11.74)	61.69 (7.83)

^1^ SNAP: Supplemental Nutrition Assistance Program. ^2^ WIC: Special Supplemental Nutrition Program for Women, Infants, and Children.

**Table 2:** FAST scores by client sociodemographic characteristics.

	FAST Score	*p*
Age (in years)		0.489
18–35	66.8 (12.9)	
36–55	63.7 (9.48)	
56+	62.0 (10.6)	
Sex		0.23
Male	64.7 (10.7)	
Female	61.7 (10.1)	
Race		0.247
Black/African Am.	62.7 (10.8)	
Others	65.6 (5.4)	
Household Size (Number of people)		0.101
1–2	65.5 (8.46)	
3–4	60.0 (13.0)	
5–6	60.9 (9.57)	
Number of Children in household		0.238
0	64.1 (8.65)	
1–2	60.0 (11.6)	
3+	65.6 (14.0)	
SNAP		0.147
Yes	64.6 (8.81)	
No	61.0 (12.0)	
WIC		<0.05
Yes	51.5 (12.1)	
No	63.7 (9.97)	
Marital Status		0.909
Married	60.5 (4.46)	
Unmarr. w/part.	64.0 (8.12)	
Unmarr. w/o part.	63.3 (11.6)	
Other	61.6 (4.74)	
Employment		0.606
Empl. 30+ h/wk	62.5 (14.5)	
Empl. <30 h/wk	56.7 (11.2)	
Seasonally empl.	65.2 (NA)	
Unemployed	64.2 (10.8)	
Retired	60.8 (9.85)	
Disabled	65.1 (9.36)	
High blood pressure		0.657
Yes	62.6 (8.88)	
No	63.7 (12.6)	
Diabetes		0.905
Yes	63.3 (8.08)	
No	63.0 (11.1)	
Obesity		0.361
Yes	60.2 (13.6)	
No	63.5 (9.85)	
Cancer		0.716
Yes	64.5 (5.37)	
No	62.9 (10.7)	

**Table 3:** FAST scores by food pantry size and distribution methods.

	Distribution Methods			*p*
Pantry Size	Client-Choice	Traditional		
Overall	61.7	63.9		0.371
Small	66.8	64.3		0.40
Medium	57.6	67.7		<0.01
Large	60.6	62.2		0.71
	Pantry size			
Distribution methods	Small	Medium	Large	
Overall	65.5	62.6	61.7	0.430
Client-choice	66.8	58.0	60.6	<0.05
Traditional	64.3	67.7	62.2	0.474

**Table 4:** Differences in GWS by pantry size and distribution methods.

Categories	Total (*n* = 74)	Food Pantry Size				Food Distribution Methods		
		Small (*n* = 21)	Medium (*n* = 20)	Large (*n* = 33)	*p*	Traditional (*n* = 45)	Client-Choice (*n* = 29)	*p*
FAST score (mean (SD))	63.04 (10.38)	65.49 (6.79)	62.62 (7.39)	61.75 (13.37)	0.430	63.92 (11.74)	61.69 (7.83)	0.371
Fresh Fruits and Vegetables (mean (SD))	0.17 (0.21)	0.30 (0.29)	0.14 (0.17)	0.10 (0.12)	<0.01 **	0.18 (0.24)	0.14 (0.15)	0.342
Processed Fruits and Vegetables (mean (SD))	0.22 (0.16)	0.14 (0.15)	0.30 (0.13)	0.22 (0.17)	<0.01 **	0.23 (0.19)	0.20 (0.11)	0.343
Whole Grains (mean (SD))	0.04 (0.05)	0.06 (0.06)	0.04 (0.05)	0.03 (0.04)	0.177	0.04 (0.06)	0.05 (0.05)	0.407
Non-Whole Grains (mean (SD))	0.08 (0.11)	0.03 (0.04)	0.08 (0.09)	0.10 (0.15)	0.064	0.06 (0.14)	0.11 (0.06)	0.093
Beverages (mean (SD))	0.01 (0.04)	0.05 (0.07)	0.00 (0.00)	0.00 (0.00)	<0.001 ***	0.00 (0.00)	0.04 (0.06)	<0.001 ***
Desserts and Snacks (mean (SD))	0.06 (0.12)	0.03 (0.04)	0.01 (0.02)	0.10 (0.17)	<0.05 *	0.06 (0.16)	0.04 (0.03)	0.421
Dairy (mean (SD))	0.03 (0.06)	0.01 (0.01)	0.00 (0.00)	0.06 (0.08)	<0.001 ***	0.03 (0.08)	0.03 (0.04)	0.941
Vegetable Protein (mean (SD))	0.03 (0.06)	0.05 (0.08)	0.00 (0.01)	0.04 (0.05)	<0.01 **	0.04 (0.06)	0.03 (0.05)	0.806
Meat, Poultry, Fish and Eggs (mean (SD))	0.15 (0.13)	0.03 (0.05)	0.16 (0.07)	0.21 (0.14)	<0.001 ***	0.17 (0.15)	0.11 (0.08)	0.082
Highly Processed Meat	0.02 (0.04)	0.01 (0.03)	0.00 (0.00)	0.03 (0.05)	<0.05 *	0.02 (0.04)	0.01 (0.04)	0.805
Mixed Meals and Side Dishes (mean (SD))	0.17 (0.16)	0.29 (0.10)	0.19 (0.17)	0.07 (0.12)	<0.001 ***	0.16 (0.17)	0.19 (0.14)	0.428
Condiments, Baking and Cooking (mean (SD))	0.03 (0.06)	0.01 (0.02)	0.07 (0.09)	0.02 (0.04)	<0.01 **	0.02 (0.03)	0.06 (0.08)	<0.01 **
Infant Formula (mean (SD))	0.00 (0.01)	0.00 (0.00)	0.00 (0.00)	0.00 (0.01)	0.544	0.00 (0.00)	0.00 (0.01)	0.215

*: *p* < 0.05; **: *p* < 0.01; ***: *p* < 0.001.

## Data Availability

Data available on request due to privacy.
